# Multidisciplinary teams in the clinical care of fibrotic interstitial lung disease: current perspectives

**DOI:** 10.1183/16000617.0003-2022

**Published:** 2022-09-07

**Authors:** Vincent Cottin, Fernando J. Martinez, Vanessa Smith, Simon L.F. Walsh

**Affiliations:** 1 Louis Pradel Hospital, Reference Centre for Rare Pulmonary Diseases, Hospices Civils de Lyon, Lyon, France; 2 Claude Bernard University Lyon 1, UMR754, INRAE, Member of OrphaLung, RespiFil, Radico-ILD and ERN-LUNG, Lyon, France; 3 Dept of Medicine, Cornell University, New York City, NY, USA; 4 Dept of Rheumatology, Ghent University Hospital Dept of Internal Medicine, Ghent, Belgium; 5 Dept of Internal Medicine, Ghent University, Ghent, Belgium; 6 Unit for Molecular Immunology and Inflammation, VIB Inflammation Research Centre (IRC), Ghent, Belgium; Member of ERN ReCONNET (rare connective tissue diseases); 7 National Heart and Lung Institute, Imperial College London, London, UK

## Abstract

Multidisciplinary team (MDT) meetings, involving the integrated collaboration of healthcare professionals, are increasingly used in clinical practice to inform the diagnosis and treatment of interstitial lung diseases (ILDs). Over time, the assessment of patients with ILD has transitioned from discussions among clinicians, radiologists and pathologists to the inclusion of a broader range of clinical data and specialist expertise. Studies have shown that a multidisciplinary approach can have many benefits for the clinical care of patients with ILD by improving the diagnostic confidence for different ILDs and guiding treatment decisions. The utility of MDT discussions for diagnosis, monitoring disease progression and management decisions, will need to be considered based on how it is best positioned in the diagnostic and therapeutic process, as well as the practicality and challenges of its use. There are also uncertainties and heterogeneity concerning the optimal practices of MDT meetings in ILD care. In this review, we describe recent developments refining the approach to MDTs in clinical practice, including who should be involved in the MDTs, when it is most needed, their use in patient management, challenges in their implementation, and ongoing controversies in the field that need further research.

## Abstract 2

**Due to the complexity of fibrotic ILDs, multidisciplinary teams are instrumental in optimising the diagnosis and management of patients.** https://bit.ly/3R9pZ6R

## Introduction

Interstitial lung diseases (ILDs) are heterogeneous disorders with different causes, clinical manifestations and treatment options. Idiopathic pulmonary fibrosis (IPF) is the most common fibrotic ILD and is characterised by a radiological and/or histological pattern of usual interstitial pneumonia (UIP) and progressive fibrosis [1]. Fibrotic ILDs are also associated with autoimmune and connective tissue diseases (CTDs), sarcoidosis and fibrotic hypersensitivity pneumonitis (HP). While fibrosis in these diseases may not always be progressive, a proportion of patients manifest progressive pulmonary fibrosis (PPF) [2], which shares a disease course similar to IPF and is characterised by high mortality and decline in lung function [3–5]. The similar clinical presentations of these varied conditions can result in difficulties obtaining a definitive diagnosis, which can impact clinical decision-making and the timely provision of appropriate treatments [1].

Early guidelines set by the American Thoracic Society (ATS)/European Respiratory Society (ERS) committee recommended the use of multidisciplinary teams (MDTs) as a gold standard for diagnosing patients with IPF [6, 7]. Subsequent expansion of these guidelines further strengthened the role of MDTs [8]. Several studies have shown that a comprehensive assessment of clinical, radiological and pathological data by ILD experts can improve diagnostic agreement [9–12]. MDTs also draw information from a range of specialities to interpret available evidence for reaching a consensus diagnosis [13]. While MDTs in ILD have mainly focussed on diagnosis, there is also evidence that MDT discussions may benefit management and follow-up of patients. Within other fields, such as oncology, MDTs are a key component in patient care and the treatment decision process. With the emergence of new therapeutic interventions being used to treat fibrotic ILDs, there is a need for more accurate diagnoses and better predictors of longitudinal disease behaviour in specific ILDs to further optimise and tailor individual patient treatment plans [14].

Multidisciplinary approaches may vary from one group to another, which may depend on the availability of local resources and differences in healthcare systems of different countries. In some settings, a multidisciplinary diagnosis of IPF is a condition for the reimbursement of antifibrotic treatments [15, 16]. Uncertainties regarding the optimal MDT structure remains a challenge [17]. Data comparing different approaches could help improve the framework around MDT practice and possibly lead to harmonisation of some of its aspects; however, there is a lack of formal criteria for the organisation and validation of MDTs [17].

MDTs are continually evolving in response to patient needs and therapeutic developments, and this review will outline current perspectives on their use in the clinical care of ILDs. A podcast summarising this review article is available at: www.globalmedcomms.com/respiratory/Cottin/MDTinongoingmanagementofILDs

## History of MDTs in ILD

In 2002, a statement by the ATS and ERS advocated an integrated approach involving clinical, radiological and pathological data for the diagnosis of idiopathic interstitial pneumonia (IIP) [6]. A landmark study by Flaherty *et al*. [12] involving 58 patients with ILD showed that a multidisciplinary approach involving clinicians, radiologists and pathologists increased the confidence level for a given diagnosis. In this study, MDT participants were provided with information in a stepwise manner and asked to record their diagnostic impression and level of confidence when making a consensus diagnosis for patients with suspected IIP. This incremental process demonstrated that discussions and exchange of information between clinicians, radiologists and pathologists improved the overall interobserver agreement and confidence level for diagnosing ILD. In addition, histopathological findings led to improved agreement between clinicians and radiologists. This study underscored the importance of a multidisciplinary approach for improving the quality of how physicians diagnose ILD. Subsequently, MDTs have been increasingly recognised for diagnosing fibrotic ILDs in several consensus statements and guidelines that outline recommendations for their format and use (see table 1).

**TABLE 1:** Guideline recommendations for the use of multidisciplinary teams (MDTs) in fibrotic interstitial lung diseases (ILDs)

**ILD**	**Published guidelines (year)**	**Format**	**Uses**
**ILD**	British Thoracic Society in collaboration with the Thoracic Society of Australia and New Zealand and the Irish Thoracic Society (2008) [85]	Minimum MDT participants: expert clinicians, pathologists, and radiologists Patients with ILD should have access to an MDT based in a regional centre with expertise in ILD	The diagnosis of HP requires a high index of suspicion, and a multidisciplinary approach is essential in difficult cases
**IPF**	French practical guidelines (2022) [86]	Minimum MDT participants: pulmonologists, radiologists and pathologists with experience in the field of interstitial pneumonia MDT discussion should take place in an expert or specialised centre	The decision to perform a biopsy is taken during an MDT discussion after a risk assessment based on age, the functional impact of the disease, the existence of comorbidities and the ILD evolution MDT is used for diagnostic decision-making, and therapeutic management is also discussed
	Haute Autorité de Santé guidelines (2021) [87]	Minimum MDT participants: pulmonologists, radiologists and pathologists with experience in the field of interstitial pneumonia	When CT scan shows an aspect of probable UIP or UIP, the diagnosis can be retained in MDD without resorting to lung biopsy
	ATS/ERS/JRS/ALAT (2018) [8]	Minimum MDT participants: pulmonologist, radiologist and pathologist (and rheumatologist on a case-by-case basis) The method of interaction is decided by the involved clinicians and could be face to face, or using telephone, email, text or fax	MDD can be used to reach a confident diagnosis of IPF when HRCT and histopathological patterns are discordant MDD can be used to reclassify to a more specific diagnosis when HRCT and histopathological patterns are both indeterminate for IPF The decision to perform SLB should be made by experienced clinicians in an MDT
	Fleischner Society (2018) [23]	Key features of MDT process: Not all patients with IPF require MDD and should be used when patients are not adequately categorised based on evidence base. MDT may be useful to: • decide whether to perform a biopsy if imaging and clinical features do not provide enough diagnostic confidence • review the clinical, imaging and pathological features after a biopsy • review cases where the longitudinal course is discordant with diagnosis Minimum MDT participants: clinician, radiologist and pathologist; rheumatologists, occupational physicians and geneticists may be important in specific cases Diagnosis should be clearly communicated on whether formal IPF diagnostic criteria were reached or if clinical reasoning was used to obtain a working diagnosis	When is MDD necessary in the context of suspected IPF? Where the clinical context and/or the CT pattern are indeterminate outcome of MDD will be a decision whether to perform additional clinical evaluation and/or BAL and/or diagnostic biopsy After biopsy is performed, to integrate the clinical, imaging and histological features When diagnostic tissue is not available, to consider a working diagnosis of IPF Goals of multidisciplinary conference include: diagnosis, management plan, review of disease progression
		Desirable features of multidisciplinary conference: Frequency of meeting: weekly/monthly Nature of meeting: direct contact/telemedicine Participants: clinician, radiologist, pathologist. If not experienced in ILD, linkage to an experienced group is needed Documentation: first-choice diagnosis, realistic differential diagnoses. Recommendation for additional diagnostic test Communication: final multidisciplinary diagnosis recorded in case notes and communicated in discharge statement	
	NICE guidelines (2017) [24]	Minimum MDT participants: After clinical evaluation, PFTs and CT: physician, radiologist, specialist nurse, MDT coordinator When considering BAL, TBB, SLB: include thoracic surgeon and histopathologist When considering results of BAL, TBB, SLB: include histopathologist	If the MDT cannot make a confident diagnosis from clinical features, lung function and radiological findings, consider: BAL, TBB and/or SLB, with the agreement of the thoracic surgeon
**HP**	ATS/ERS/JRS/ALAT practice guideline (2020) [29]	If patients cannot be diagnosed with HP with high confidence (based on identified exposure, typical HP pattern on HRCT, and BAL lymphocytosis) they should undergo an MDT discussion that includes a pulmonologist, chest radiologist and pathologist (if transbronchial lung biopsies were performed at time of BAL) If the patient has a culprit exposure, initial assessments include HRCT and BAL followed by MDD If the patient has no culprit exposures, and is a male former smoker and >60 years old, then the 2018 ATS/ERS/JRS/ALAT guidelines for IPF diagnosis should be applied (HRCT scan followed by an MDD)	
	CHEST guideline and expert panel report (2021) [88]	If the inciting antigen is suspected to be related to an occupational exposure, an occupational medicine specialist and an environmental hygienist should be included in the multidisciplinary diagnostic workup If the biopsy is indeterminate for HP or compatible with HP, a provisional diagnosis can be made after careful consensus MDD	
**CTD-ILD**	Thoracic Society of Australia and New Zealand (2021) [89]	Face-to-face discussion by treating clinician with respiratory physicians, a radiologist and, if relevant, a histopathologist	
**Fibrotic ILDs**	Canadian Thoracic Society (2017) [90]	Should involve expert respirologists, radiologists and pathologists	MDT discussions are an iterative process, and patients should be re-reviewed if new information becomes available MDTs should review patients before use of disease-specific treatments
**PPF** ^#^	Erice ILD working group (2020) [53]		Patients should be reassessed by MDTs at regular intervals to ensure the early identification of patients meeting the definition of PPF For patients with PPF, treatment with antifibrotic therapy and immunosuppression should involve a case-by-case assessment by an MDT

ALAT: Latin American Thoracic Society; ATS: American Thoracic Society; BAL: bronchoalveolar lavage; CT: computed tomography; CTD: connective tissue disease; ERS: European Respiratory Society; HP: hypersensitivity pneumonitis; HRCT: high-resolution computed tomography; IPF: idiopathic pulmonary fibrosis; JRS: Japanese Respiratory Society; MDD: multidisciplinary discussion; NICE: National Institute for Health and Care Excellence; PFT: pulmonary function test; PPF: progressive pulmonary fibrosis; SLB: surgical lung biopsy; TBB: transbronchial biopsy; UIP: usual interstitial pneumonia. ^#^: referred to as progressive fibrosing ILD in original publication.

In the past two decades, the longitudinal care needs of patients with ILD have resulted in specialist ILD centres to manage and support patients [18]. Results from a Delphi survey and patient focus group analysis focusing on the essential components of an ILD clinic have proposed multidisciplinary conferences as one of the main components [19]. In addition, the European Commission has established European reference networks to facilitate multidisciplinary discussions for rare lung diseases through networks involving healthcare providers across hospitals in Europe [20].

## Disease characteristics and the roles within an MDT

Determining the aetiology of pulmonary fibrosis requires the comprehensive assessment of disease-specific factors, including a thorough history of extrapulmonary signs, exposures and medication use, serological testing, lung function testing, high-resolution computed tomography (HRCT) imaging and lung biopsy [1, 21]. Although genetic testing is not widely used for diagnosis, clinicians should routinely obtain family history of ILD and manifestations, as this may be suggestive of alterations in telomere-related genes [22]. Due to the complexity of disease presentation in cases of fibrotic ILDs, evaluating clinical evidence requires a multidisciplinary discussion involving expert specialists in the diagnostic pathway.

**FIGURE 1:**
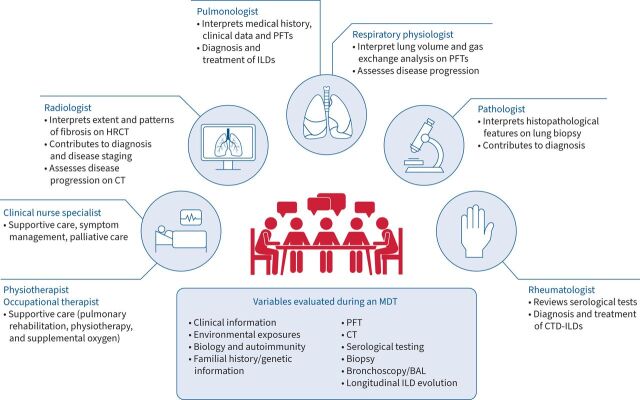
Example of roles in an multidisciplinary team (MDT) for diagnosis and management. BAL: bronchoalveolar lavage; CT: computed tomography; CTD: connective tissue disease; HRCT: high-resolution computed tomography; ILD: interstitial lung disease; PFT: pulmonary function testing.

The ATS/ERS consensus statements and 2017 Fleischner Society white paper suggest that MDTs should, as a minimum, include a pulmonologist, a radiologist and a pathologist to integrate different clinical data before forming a final diagnosis [6, 7, 23]. The National Institute for Health and Care Excellence guidelines also stipulate a minimum composition of the MDT for diagnosing IPF, including a specialist radiologist, histopathologist, clinician and clinical nurse specialist [24]. A collective analysis of various studies by Furini *et al*. [13] found that the most frequently involved physicians in an MDT were thoracic pathologists (23/29 studies), thoracic radiologists (26/29 studies) and pulmonologists (29/29 studies). However, a range of other specialities was also reported in several studies, which include the integration of rheumatologists, clinical nurse specialists, occupational therapists, cardiothoracic surgeons and lung transplantation teams. The composition of members may depend on the purpose of an MDT (figure 1). It could be argued that the true purpose of an MDT in ILD is ultimately to make a diagnosis; as such, the core group of specialists would involve a pulmonologist and radiologist, with the inclusion of other specialities (pathologists and rheumatologists) considered on a case-by-case or centre-by-centre basis [25]. However, the scope of MDTs in ILD has expanded to encompass other specialists who may benefit the dynamic discussions around diagnosis, monitoring disease progression and management decisions. For example, rheumatologists and transplant surgeons are also key contributors in an MDT to provide management recommendations for CTD-ILDs and lung transplantation, respectively, although the availability of these speciality groups to participate in a meeting is a limitation [26]. Pulmonologists may be best placed to take part in monitoring and management discussions as they directly consult with the patient, along with integrating clinical data. Additional noncore members may also advise on the supportive care for patients (palliative care and transplant teams) but may not need to attend regular MDT meetings. Assessing patient comorbidities and preferences is especially important considering that treatment options are often associated with adverse events that may affect a patient's quality of life [26].

Regarding pathology, guidelines recommend that MDTs should first evaluate the available evidence collected based on typical clinical and HRCT features before evaluating whether a biopsy is necessary [7, 23]. If a biopsy is performed, a second MDT discussion may help to evaluate its results. It is important that the sample is adequately obtained to provide diagnostic information, and MDT discussions may be useful in determining the most appropriate area for tissue sampling and the type of biopsy used [16]. There is accumulating evidence that transbronchial lung cryobiopsy is a safe method for histopathological diagnosis. The decision to use it over surgical lung biopsy is evolving, taking into account the institutional expertise of the operator and the pathologist, with recent ATS guidelines recommending its use for evaluating fibrotic HP and IPF [2, 27–29]. In this regard, the multidisciplinary discussion integrates the available information, including local experience with different techniques and their local availability, to propose the best approach to diagnosis.

The involvement of a rheumatologist is crucial in the assessment of CTD-ILDs [15] but may not be mandatory in every ILD MDT discussion. In the care pathway of a rare CTD, if a patient develops a manifestation of ILD during an established CTD, rheumatologists are commonly involved. On the other hand, if the pulmonary involvement is the first manifestation, with CTD symptoms appearing later, the patient will logically be discussed in an ILD MDT, where the pulmonologist will convene with a rheumatologist. Current guidelines suggest that rheumatologists could be involved on a case-by-case basis, and patients should be referred to a rheumatologist when showing positivity from serological tests (*e.g.* autoantibody tests) or presenting clinical manifestations suggesting an underlying rheumatological disease [8]. In some cases, it may be difficult to distinguish patients with IIP from those with CTD-ILD, as with interstitial pneumonia with autoimmune features, which does not fit classical CTD criteria [13]. Rheumatology input may be particularly valuable during the examination of the patient and history taking, where subtle signs of a CTD may be elicited that might otherwise be missed [30, 31]. Additionally, the rheumatologist may aid in management decisions for rare CTDs with a heterogeneous course of associated ILD [32, 33].

## What cases are likely to benefit from MDT characterisation?

It should be noted that not all cases of suspected ILD require MDT characterisation. Since the initial clinical assessment can determine a diagnosis of IPF based on radiological evidence of UIP and clinical history, or confirm CTD-ILD through computed tomography and serological testing, an MDT may not always be required. Given the risks of additional invasive procedures, patients for whom this approach is being considered may benefit from MDT discussion before and after a biopsy. Therefore, an MDT would be needed in only a proportion of cases with difficult diagnostic and therapeutic dilemmas.

A proportion of ILDs remains unclassifiable after initial assessment [34]. Methods to classify different types of fibrotic ILD include terminology based on diagnostic confidence (confident diagnosis, provisional diagnosis and unclassifiable), as suggested by a working group led by Ryerson *et al*. [35]. One of the roles of an MDT is to assess the level of confidence in the diagnosis, to propose a first-choice diagnosis and to identify alternative diagnoses that can be considered. When there is no clear diagnosis, patients could be managed based on a “working diagnosis” – a non-definite diagnosis based on sufficient confidence and clinical reasoning. This accounts for disease behaviour over time, potentially requiring future MDTs at regular intervals to evaluate clinical information and HRCT evolution [30, 36, 37]. Recently, a Bayesian diagnostic approach has been proposed to integrate the clinician's clinical judgement into an IPF diagnosis, where the clinical features and the disease behaviour (*e.g.* observed outcome of disease with or without therapy) are integrated together with radiological and pathological features when available [37]. This concept especially helps to differentiate IPF from other fibrotic ILDs and may obviate the need for histopathology in a number of cases.

The presence of a UIP pattern on HRCT is a key component in the diagnostic process of IPF. In patients with a clear UIP pattern and no signs of antigen, environmental exposure or a systemic disorder, a diagnosis of IPF may not require an MDT [7]. Guidelines suggest that an MDT can be especially relevant in cases where the radiological pattern is not UIP, and when it is discordant from the pathological pattern (*e.g.* HRCT is inconsistent with UIP, but the histopathology result indicates UIP) [38].

Although UIP is commonly associated with IPF, it is also observed in other ILDs, such as fibrotic HP and rheumatoid arthritis-associated ILD [39]. Patients with fibrotic HP can have similar histopathological and radiological manifestations to those with IPF, including a UIP-like pattern of fibrosis on lung biopsy, requiring the assessment of exposure-related symptoms and HRCT features to distinguish fibrotic HP from IPF [29, 40]. A potential exogenous origin may still be causative in the case of definite UIP, and a careful assessment for exposure (history, standardised questionnaire, serology) and bronchoalveolar lavage may be instructive [27, 29, 41]. The risks and invasiveness of performing a biopsy can deter patients from its use when it is needed for the confirmation of a UIP pattern. In the absence of biopsy, key multidisciplinary information that can impact the “classic UIP” found on HRCT includes age, gender, decline in lung function and smoking history, which can determine a working diagnosis of IPF or provide an alternative diagnosis of a different ILD [42, 43]. In some cases, patients initially considered not to have IPF who fail to respond to immunosuppressive treatment experience an irreversible, fibrosing phenotype of disease progression, leading clinicians to revisit the diagnosis and reconsider the diagnosis of IPF in an MDT discussion based on disease behaviour.

Multiple groups have highlighted the effect of MDT discussion for confirming or changing the preliminary diagnosis [42, 44]. De Sadeleer *et al*. [45] demonstrated that an MDT changed the diagnosis in 41.9% of patients, as well as suggesting first-choice diagnosis in 80.5% of cases previously considered unclassifiable. In another study, ILD diagnoses were changed in 53% of patients following MDT discussion, resulting in an increase in the proportion of patients diagnosed with CTD-ILD (from 10% to 21%) and hypersensitivity pneumonitis (from 3% to 16%) [46]. Overall, this highlights that as well as their role in diagnosing IPF, MDTs play a role in the broader discussion around non-IPF ILD and can help expand the expertise and confidence for making an alternative diagnosis.

## Impact of MDTs on the management of fibrotic ILDs

### Accuracy of diagnosis on management

Several studies have investigated the potential benefits of MDT diagnosis on improving inter-observer agreement or leading to a change in the final diagnosis or management recommendation using a “pre-MDT” and “post-MDT” study design (table 2). The accuracy of MDT diagnosis of ILD is an important step that leads to the development of management strategies for specific ILDs, especially where the underlying cause of the disease is rare and difficult to assess [17]. For example, identifying inciting antigens can improve outcomes for patients diagnosed with fibrotic HP [47]. A global study evaluating ILD diagnostic practices across different countries found that patient management was discussed in 94.9% of MDT diagnostic meetings in centres [48].

**TABLE 2:** Summary of the impact of multidisciplinary team (MDT) discussion in interstitial lung disease (ILD) diagnosis and treatment

**Author (year) [ref.]**	**Number of patients**	**MDT members**	**Results for diagnosis**	**Results for treatment**
**Flaherty *et al.* (2004) [12]**	58	Three pulmonologists, two radiologists and two pathologists	Increased level of interobserver agreement in suspected IIP Pathologists changed their original diagnosis in 19% of cases once the clinical and radiological data became available	No information provided
**Flaherty *et al.* (2007) [9]**	39	Clinicians, radiologists and pathologists	An interactive approach between clinicians, radiologists and pathologists improved interobserver agreement at both community and academic sites Interobserver agreement was higher in academic centres (κw=0.55–0.71) than within community centres (κw=0.32–0.44)	No information provided
**Mittoo *et al.* (2009) [91]**	114	Evaluation at an ILD clinic	34 (30%) patients in an interdisciplinary ILD programme were found to have CTD • 17 patients presented with an established CTD, and 17 patients were newly diagnosed following their evaluation at the ILD clinic	No information provided
**Castelino *et al.* (2011) [92]**	50	Concurrent evaluation of patients with ILD by a pulmonologist and a rheumatologist	Of the patients with a final diagnosis of CTD-ILD, 28% were referred with a diagnosis of IPF 36% of CTD-ILD patients had their diagnosis changed to an alternate CTD-ILD	IPF: change in therapy in 27% of patients (4/15) CTD-ILD: changes in therapy in 80% of patients (20/25) (mostly to a combination of CSs with immunomodulatory agent)
**Walsh *et al.* (2016) [10]**	70 patients included in the final study cohort	Clinician, pathologist and radiologist	Inter-MDT meeting agreement on diagnostic likelihoods was good for IPF (κw=0.71) and CTD-ILD (κw=0.73), moderate for NSIP (κw=0.42) and fair for HP (κw=0.29)	No information provided
**Chaudhuri *et al.* (2016) [42]**	318	Two respiratory physicians, one radiologist, one pathologist, one specialist nurse, one MDT coordinator and one ILD pharmacist	Consensus diagnosis made in 57/75 (76%) of unclassifiable ILDs IPF diagnoses were correct in 50/107 (47%) cases and incorrect in 57/107 (53%) cases Other ILD diagnoses were correct in 91/136 (67%) cases and incorrect in 45/136 (33%) cases	IPF: MDT discussion resulted in a change of treatment for 50% of patients (53/107) (stopping immunosuppressant therapies and initiating pirfenidone) Other ILDs: MDT discussion resulted in a change of treatment in 39% (53/136) of cases (starting or discontinuing immunosuppressant therapies)
**Tomassetti *et al.* (2016) [28]**	117	Two clinicians, two radiologists and two pathologists	The overall inter-observer agreement in IPF diagnosis was similar for both BLC (overall kappa: 0.96) and SLB (overall kappa: 0.93) After the addition of histopathological information in an MDT: • 17% of cases in the BLC group were reclassified as IPF • 19% of cases in the SLB group were reclassified as IPF	No information provided
**Jo *et al.* (2016) [46]**	90	One pulmonologist, one rheumatologist, one radiologist, one pathologist and one immunologist	CTD-ILD: diagnoses increased from 10% to 21% HP: diagnoses increased from 3% to 16% IPF: 7 patients with unclassifiable ILD or NSIP had diagnosis changed to IPF	Recommendation for CS use decreased from 36% to 28% (p=0.26) Recommendation for nonsteroid immunosuppression increased from 10% to 17% (p=0.16) Recommendation for pulmonary vasodilators increased from 0% to 4% (p=0.046) Recommendation for antifibrotic therapy increased from 3% to 21% (p=0.0002) Recommendation for clinical trials increased from 0% to 3% (p=0.08) Recommendation for oxygen increased from 6% to 10% (p=0.046)
**Burge *et al.* (2017) [93]**	71	Pathologists, radiologists, clinicians and a clinical nurse specialist	MDT changed the original histological diagnoses in 30% of patients (95% CI 19.3–41.6) • UIP diagnosis: 21 patients identified in MDT compared with 16 in the histology report • HP: eight patients identified in MDT compared with two in histology report Strengthened diagnoses from probable to confident in 17% of patients (95% CI 9.1–27.7)	No information provided
**De Sadeleer *et al*. (2018) [45]**	938	Experts in pulmonology, radiology and histopathology (and other specialities when needed)	MDT discussions provided a definite diagnosis in 80.5% of the cases MDT discussions changed the diagnosis in 191 patients with a pre-MDD diagnosis (41.9%) Sarcoidosis (n=82): 62% confirmed by MDT, 6% changed by MDT Other ILD (n=31): 39% confirmed by MDT, 23% changed by MDT IPF (n=326): 38% confirmed by MDT, 6% changed by MDT CTD-ILD (n=60): 27% confirmed by MDT, 20% changed by MDT Drug-/exposure-related ILD (n=42): 26% confirmed by MDT, 21% changed by MDT Non-ILD (n=65): 22% confirmed by MDT, 37 changed by MDT HP (n=77): 21% confirmed by MDT, 31% changed by MDT COP (n=17): 18% confirmed by MDT, 18% changed by MDT RB-ILD/DIP (n=22): 14% confirmed by MDT, 18% changed by MDT iNSIP (n=33): 12% confirmed by MDT, 27% changed by MDT	No information provided
**Levi *et al.* (2018) [94]**	60	MDT of pulmonologists, radiologists, pathologists and rheumatologists	Rheumatologist assessment following routine MDT diagnosis led to: 21% of IPF diagnoses reclassified as CTD Number of CTD-ILD cases with autoimmune features increased by 77%	No information provided
**Biglia *et al.* (2019) [15]**	150	Two pulmonologists, one chest radiologist, one rheumatologist, one surgeon and one histopathologist	Total: 42% of diagnoses were revised between pre-MDD and post-MDD, leading to a significant reduction in unclassifiable ILD IPF: diagnoses increased from seven to 35 cases HP: diagnoses increased from 11 to 20 cases Unclassifiable: diagnoses decreased from 56 to 15 cases (p<0.0001)	MDD led to a change or initiation of treatment in 54% of cases
**Fujisawa *et al.* (2019) [82]**	524	One pulmonologist, one radiologist and one pathologist	MDT resulted in a change in diagnosis for 219 patients (47%) IPF (n=227): 59 cases (26%) were reclassified as unclassifiable IIPs, while 151 (67%) were confirmed as IPF iNSIP: 42 cases (43%) were recategorised as unclassifiable IIPs, 17 (17%) as IPF and three (3%) as CTD-ILD	No information provided
**Grewal *et al.* (2019) [84]**	209 internal patients 91 external patients	Pulmonologists, radiologists and pathologists	Internal patients IPF (n=54): diagnosis changed in 33% of patients iNSIP (n=4): diagnosis changed in 75% of patients HP (n=21): diagnosis changed in 14% of patients CTD-ILD (n=15): diagnosis changed in 20% of patients Other ILD (n=11): diagnosis changed in 27% of patients Unclassifiable (n=104): diagnosis changed in 43% of patients External patients IPF (n=12): diagnosis changed in 42% of patients iNSIP (n=6): diagnosis changed in 100% of patients HP (n=10): diagnosis changed in 40% of patients CTD-ILD (n=6): diagnosis changed in 50% of patients Other ILD (n=10): diagnosis changed in 40% of patients Unclassifiable (n=47): diagnosis changed in 36% of patients	After MDT review, treatment was initiated in 45% of patients on no ILD therapy and treatment was changed in 45% of patients on ILD therapy Internal patients Antifibrotic (n=7): treatment changed in 14% of patients CS (n=14): treatment changed in 86% of patients SSA (n=9): treatment changed in 11% of patients CS and SSA (n=12): treatment changed in 17% of patients No treatment (n=167): treatment changed in 49% of patients External patients Antifibrotic (n=2): treatment changed in 50% of patients CS (n=11): treatment changed in 73% of patients SSA (n=2): treatment not changed in patients CS and SSA (n=1): treatment changed in 100% of patients No treatment (n=75): treatment changed in 36% of patients
**Han *et al.* (2019) [54]**	56	Four pulmonologists, one radiologist and one pathologist	Follow-up data changed the original MDD diagnosis in 10.7% (6/56) of patients and changed the consensus level in 25% (14/56) of patients	No information provided
**Tirelli *et al.* (2020) [95]**	119	Six pulmonologists, three rheumatologists, two radiologists and one pathologist	A multidisciplinary approach was useful for identifying underlying CTD-ILD/IPAF in patients referred for ILD: • 15% had underlying CTD • 33% had IPAF • 52% had ILD without detectable CTD	No information provided
**Ageely *et al.* 2020 [96]**	126	Respirologists, one registered nurse, one thoracic pathologist and one thoracic radiologist	MDD altered the diagnosis in 37% (47/126) of cases IPF: diagnoses increased from 24 to 34 cases HP: diagnoses increased from 20 to 21 cases Sarcoidosis: diagnoses decreased from four cases to one case Nonspecified ILD: diagnoses decreased from 52 to 0 cases IPAF: diagnoses increased from 0 to five cases Unclassifiable ILD: diagnoses increased from 0 to 27 cases No ILD: diagnoses increased from 0 to six cases	Management was changed in 39% (50/126) of patients Among concordant pre-MDT and post-MDT diagnoses, management was changed in 46% (24/52) of cases
**De Lorenzis, 2020 [97]**	151	Minimal attendance: two pulmonologists, two chest radiologists and one pathologist Extended MDT meeting: included two extra rheumatologists	The agreement between rheumatologists and pulmonologists was moderate for the detection of autoimmunity test positivity (κw=0.475, p<0.001) and family history of SARD (κw=0.491, p<0.001), and fair for the identification of extrapulmonary symptoms (κw=0.225, p=0.064) or routine laboratory abnormalities consistent with SARD (κw=0.101, p=0.081) The agreement between rheumatologist and extended MDT for the identification of ILD progression was moderate (κw=0.436, p<0.001)	Therapeutic strategy changed in 72 patients (55.5%) following the extended MDT (with rheumatologists) Immunosuppressive drug (cyclophosphamide, azathioprine or mycophenolate mofetil with or without prednisone) prescribed in 50/72 (40.3%) patients Other immunosuppressants (rituximab or tocilizumab) prescribed in 13/72 (10.3%) patients Antifibrotic treatment (nintedanib or pirfenidone) prescribed in 9/72 (7.3%) patients

BLC: bronchoscopic lung cryobiopsy; CI: confidence interval; COP: cryptogenic organising pneumonia; CS: corticosteroid; CTD: connective tissue disease; DIP: desquamative interstitial pneumonia; HP: hypersensitivity pneumonitis; IIP: idiopathic interstitial pneumonia; iNSIP: idiopathic nonspecific interstitial pneumonia; IPAF: interstitial pneumonia with autoimmune features; IPF: idiopathic pulmonary fibrosis; κw: Cohen's weighted kappa; MDD: multidisciplinary discussion; NSIP: nonspecific interstitial pneumonia; RB: respiratory bronchiolitis; SARD: systemic autoimmune rheumatic disease; SLB: surgical lung biopsy; SSA: steroid-sparing agent; UIP: usual interstitial pneumonia.

The distinction between IPF and non-IPF ILDs is particularly important given the worse prognosis in IPF compared with other fibrotic ILDs. Being able to distinguish between IPF and non-IPF ILDs is critical for clinical practice, where immunosuppression may benefit non-IPF ILDs but is associated with increased risk of death and hospitalisation for patients with IPF [49, 50]. Studies have shown that management recommendations following MDT discussion can lead to changes in the use of corticosteroids and immunosuppressive therapies, nonpharmacological therapies (*e.g.* supplemental oxygen), pulmonary vasodilators and antifibrotics [15, 46], highlighting the impact that MDTs can have on the treatment of patients with suspected ILD.

### Monitoring and management of progressive disease

Different subtypes within the spectrum of fibrotic ILDs can develop PPF despite immunosuppressive therapy and the avoidance of disease-triggering stimuli. Patients with different ILDs have variable trajectories for progression, with some remaining stable and others developing inexorable progression [1]. As studies have shown that IPF and PPF may share similar pathophysiological mechanisms, it has been proposed that patients should be grouped for treatment based on shared disease behaviour, regardless of the underlying diagnosis [51]. This concept has been supported in the 2013 ATS/ERS classification, highlighting that management should be based on the most probable diagnosis after MDT discussion and consideration of expected disease behaviour [7]. Data from the INBUILD trial showed that patients with PPF other than IPF showed similar disease progression in terms of decline in forced vital capacity and mortality compared with patients with IPF in the INPULSIS trials [52]. Therefore, the correct diagnosis and follow-up of patients are needed to better define progression in fibrotic ILDs, which can have implications for therapeutic strategy [53]. Unfortunately, there has been no standardised definition for progression, and it may be difficult to predict when fibrotic ILDs will become progressive. The recent 2022 international guideline has included a definition of PPF for use in clinical practice, based on deteriorating lung function, HRCT findings and patient symptoms [2]. As disease progression can be monitored using a variety of methods, MDTs may be useful in the follow-up of patients with ILD [54]. The input of pulmonologists and serial pulmonary function tests (PFTs) is one of the main components for monitoring disease progression, and it has previously been shown that a decline in forced vital capacity of ≥10% is associated with greater mortality [55, 56]. In many university hospitals, running the PFT lab has become a subspeciality of pulmonology/physiology, with respiratory physiologists playing an increasing role in patient management through their contribution in interpreting pulmonary physiology variables and assessing disease progression. Other than lung function, the multifaceted approach for monitoring may include a combination of HRCT imaging, exercise testing and symptom evaluation [1, 57]. Serial HRCT monitoring has been evaluated in a number of studies on its ability to provide information on the evolution of ILD in patients with worsening symptoms [58]. Wider use of quantitative imaging techniques, to more accurately characterise the extent of lung fibrosis to supplement the visual assessment by radiologists, could lead to earlier identification of disease progression during routine HRCT follow-up [58, 59].

As the disease course is continually monitored, the role of MDT discussion is no longer simply a diagnostic exercise, but a discussion of the ongoing management options and evidence of ILD progression (see table 3). Based on the results of clinical trials, both nintedanib and pirfenidone were able to slow the decline in lung function compared with placebo in patients with non-IPF fibrotic ILDs [60, 61], as well as unclassifiable ILD [62]. The sequence of medications is important, as the use of immunosuppressive treatment is usually considered as first-line therapy, whereas antifibrotic treatment is initiated to manage progressive disease. In rheumatoid arthritis-associated ILD, given the need to treat both the ILD and articular disease, it has been suggested that multidisciplinary decision-making involving both pulmonologists and rheumatologists is the optimal model for making treatment decisions [63]. This can help to determine whether treatment goals should be driven by systemic disease, pulmonary disease, or both, particularly in the case of cardiopulmonary disorder co-existing with systemic disease [64]. The optimal therapeutic strategy for systemic sclerosis (SSc)-associated ILD involves the consideration of antifibrotic treatment alone or in the background of immunosuppressive therapy (primarily mycophenolate) or no therapy, based on evidence of it preserving lung function in the SENSCIS (Safety and Efficacy of Nintedanib in Systemic Sclerosis) trial in SSc-ILD [65, 66]. More recently, the anti-interleukin-6 receptor antibody tocilizumab has been approved for the treatment of SSc-ILD [67]. Given that, in some cases, patients that present with CTD cases may be assessed by a rare CTD MDT (rheumatologist, pulmonologist, cardiologist, radiologist), it is important to refer to the multidisciplinary specialised ILD clinic when appropriate.

**TABLE 3:** Use of multidisciplinary team (MDTs) in diagnosis, monitoring and management

**MDT role**	**Comments**
**Diagnosis**	Consider available evidence for diagnosing ILD Consider degree of confidence in the diagnosis If diagnosis is not confident, discuss alternative diagnoses and use of biopsy or further assessments
**Monitoring**	Consider evidence of ILD progression Discuss frequency of monitoring
**Management**	Discuss treatment goals Discuss the benefits and risks of pharmacological therapies and non-pharmacological therapies Consider referral for lung transplantation Consider patients’ health status and individualised needs Consider patients’ preferences for treatment Patients’ progress and response to therapy
	Following identification of PPF: Discuss the initiation, escalation or change of treatment

ILD: interstitial lung disease; PPF: progressive pulmonary fibrosis.

### Supportive care and lung transplantation

At a multidisciplinary specialised ILD clinic, patients are supported by a network of professionals, including specialist nurses, physiotherapists and expert physicians, as well as given access to restricted medication and enrolment into clinical trials [18]. A study evaluating factors that patients considered important in attending an ILD clinic showed that 86% valued a multidisciplinary approach in their care [68].

Beyond the initial diagnosis, MDTs can be important in supportive care by helping patients further understand their disease and allowing patients and families to make decisions in end-of-life planning. In one outpatient study, a team of respiratory care experts, nurses, respiratory therapists, physiotherapists and a dietitian helped to reduce hospitalisations by adopting an early integrated palliative approach, with a focus on early symptom management and advance care planning [69]. Previous studies investigating the unmet needs and preferences of patients and healthcare professionals have highlighted the importance of shared care and multidisciplinary collaboration in management [70–72]. A multidisciplinary approach is also considered key for lung transplantation, as a discussion between clinicians and the transplant team can help to improve outcomes before the surgery takes place [73].

## Important considerations for implementing MDTs in clinical practice

There is a need to understand best practices of MDTs to implement them in the clinical setting. Previous diagnostic guidelines and surveys have shown that there is considerable heterogeneity in MDT meeting practices [25], and there are still unresolved controversies concerning MDT practices that will need to be addressed in future studies (table 4). A recent Delphi study was conducted among ILD experts to gain consensus on the essential and desirable features of an ILD MDT meeting [74]. Obtaining high-quality HRCT scans and the presence of a radiologist were highlighted as essential, whereas a pathologist was highly desirable. Although the role of MDTs in management recommendations was considered highly desirable, this was controversial since treatment depends on individual patient factors such as frailty and personal wishes, which cannot be understood through a discussion among ILD specialists [74]. As a minimum requirement, we recommend that cases are discussed in an MDT involving at least a pulmonologist and radiologist. MDTs should discuss difficult diagnostic cases and consider the need for a biopsy, as well as the type of biopsy (cryobiopsy/surgical lung biopsy). In this setting, the participation of a pathologist or the individual to perform invasive sampling may provide key input in identifying the site of sampling. Post-biopsy results will also need to be considered in a further MDT discussion to determine the diagnosis, which can provide the basis for therapeutic decisions.

**TABLE 4:** Unresolved controversies regarding multidisciplinary team (MDT) practice

Do all cases of ILD, even the most typical ones, need to be discussed in an MDT? Should MDTs deal with management (in addition to diagnosis)? Is the presence of both a rheumatologist and a pulmonologist always required when discussing a case of CTD-ILD? What constitutes the quorum in an MDD? Which method of lung biopsy should be used? What data should be presented in an MDT? What is the frequency of an MDT meeting? What is the potential role in MDD of noncore members (*e.g.* palliative care specialist, research nurse and physiologist)? Does the MDT have a role in other scenarios, *e.g.* assessment of longitudinal disease behaviour?

CTD: connective tissue disease; ILD: interstitial lung disease; MDD: multidisciplinary discussion.

Consistent levels of agreement are not always possible through an MDT discussion. Poor inter-MDT agreement was found between different centres in diagnosing HP and nonspecific interstitial pneumonia, which may be related to the lack of diagnostic criteria for these ILD subtypes [10]. Further research is needed on shared practices among ILD MDTs, including the format of meetings, documentation of cases and meeting coordination, to reduce heterogeneity and allow for inter-MDT benchmarking [26, 74]. A standardised template of collated patient data and regular multidisciplinary conferences (ideally once every 2 weeks) can increase harmonisation and maintain expertise [19, 26, 74]. Through a multidisciplinary discussion between team members, a collaborative environment is established within which each member contributes their expertise, which can help to improve the skillset and experience of the involved clinicians. Regular attendance at MDT meetings was shown to improve the prognostic accuracy of nonacademic clinicians up to the level of experienced ILD experts [75]. In other specialities, research into the quality of group decision-making has highlighted key factors for effective teams, such as meeting management, training, leadership, teamwork and culture, and incorporating patient views [76, 77]. Understanding how the dynamics of participants within an MDT may influence decisions and the impact of other factors will help to develop strategies for effective MDTs in the care pathway for fibrotic ILDs.

There is a lack of methods to evaluate the performance of an MDT, and difficulties in designing studies to assess its characteristics. There is currently no independent reference standard to validate an MDT diagnosis against since all the diagnostic information is considered in an MDT. It has been proposed that multidisciplinary diagnosis of IPF can be validated against the course of the disease by comparing outcomes between IPF and non-IPF diagnoses [10, 75, 78]. In one study by Walsh *et al*. [10], by using outcome distinctions between IPF and other ILDS as a surrogate for diagnostic accuracy, MDT diagnosis of IPF was more prognostically accurate than IPF diagnoses made by the individual specialists of the MDT. Assessing whether management recommendations and treatment plans are being followed by patients is another potential indicator for the effectiveness of MDTs [77].

### Barriers to implementation

Not all institutions are able to have an MDT in place, and expert centres are currently the primary location for multidisciplinary assessment [18]. While MDTs facilitate a common language of communication between the specialists, the quality of an MDT may depend on the experience and expertise of the individual physicians that form it and the accuracy of the clinical information gathered [79]. The complexity of the process in diagnosing ILD, such as detecting signs of CTD-ILD and identifying environmental exposures in fibrotic HP, suggests a need for members experienced in ILD to maximise the quality of interpreting clinical data in individual patients. Other hurdles in implementing an MDT include the potential that interactions within an MDT at a personal level may create unbalanced discussions, especially when the skills and expertise of the specialists involved differ significantly. A lack of common goals, uneven levels of expertise and inexperience with technology are some of the challenges that limit integrated collaboration of MDT members [17, 80].

A review of multidisciplinary meeting practices in expert ILD centres by Jo *et al*. [46] demonstrated significant heterogeneity in terms of meeting format and organisation, suggesting that physicians should adapt to local organisations and constraints to facilitate a good interaction between speciality physicians. This article reported the results of a survey from 10 expert centres and showed that the attendant clinician was responsible for leading the meeting in 90% of the centres, documenting the outcome of the meeting 70% of the time, and had the greatest input into formulating diagnosis 60% of the time [81]. In an international multicentre study of ILD MDTs, pulmonology was almost always represented (99.7% of centres), with radiology present at most centres (91.4%); however, histopathology specialist attendance was less frequent, with approximately a third of centres (including 26% of academic ILD centres) having no regular pathologist participant. This suggests that a low proportion of cases used biopsies to establish a diagnosis [48]. The reason for the lack of pathologist participation is not clear: this may have been due to the unavailability of biopsies and/or the few cases in which they would be required to contribute. In the same study, it was found that formal MDT meetings were more likely to be held at ILD academic centres than non-ILD academic centres or nonacademic centres, and were more common in centres in countries with higher income [48].

The process of running an MDT in busy academic practices can be burdensome and time-consuming for its participants. Where this is not possible, the treating physician should assimilate the multidisciplinary factors and evidence in each case before making diagnostic and therapeutic decisions [1]. In addition, a “nonmultidisciplinary” discussion between pulmonologists, which may take place in or outside the MDT, may be important in determining management decisions and treatment options, especially for clinicians less experienced in ILD. Smaller, community-based practices may benefit most from MDT characterisation for their cases but may lack ILD experience in their group participants. Telemedicine may be an option to allow greater access to MDTs in clinical practice and could create opportunities for improved communication when diagnosing and managing patients [82]. Virtual meetings between experts and community groups can help address gaps in expertise. The use of telemedicine has been accelerated during the coronavirus disease 2019 (COVID-19) pandemic, as it provides a platform for discussion and communication despite the geographic separation of ILD experts and limited healthcare resources. However, it should be noted that telemedicine has been applied in MDT meetings before the COVID-19 pandemic, as services across Australia have utilised a hybrid virtual/in-person MDT approach in their clinical practice [83]. Telemedicine also allows the use of enhanced and secure data-sharing platforms, which can increase collaboration among specialist and general practitioners. In one study, a web-based MDT with specialists in pulmonology, radiology and pathology was conducted for 465 cases of biopsy-proven IIP, using a nationwide cloud-based integrated database and video conferencing [82]. Although the MDT format was feasible and led to better prognostic discrimination among IIPs, it is important to note that the quality of the history and physical examination available in the database may depend on the experience of each patient's attending physician in managing ILD. Patient confidentiality should also be preserved during virtual case presentation [83]. Grewal *et al*. [84] reported findings on the feasibility of MDTs for evaluating the diagnosis and management of 91 external patients remotely. The results indicated that remote MDTs could be effective in changing the diagnosis of external patients and, in a survey, the use of external review was considered satisfactory by 93% of referring pulmonologists. However, the efficacy of remote MDTs will need to be balanced with the benefits derived from traditional in-person assessments within ILD clinics, which include access to additional support from specialist nurses, support groups, patient educators and counselling [84].

## Conclusion

MDTs can play an important role in improving the quality of how physicians make diagnostic and management decisions. The practicality of running an MDT, determining when it is most needed, and its standardisation are still matters of debate. MDTs can provide significant cooperation and alignment on diagnostic evidence to support the final diagnosis of a complex case, particularly where evidence and standardised diagnostic guidelines may be lacking. Previous studies have highlighted their ability to increase interobserver agreement and establish an accurate diagnosis, which can benefit the prognostication of ILD disease course.

Although the MDT was originally developed to improve diagnostic evaluation of ILD, discussions have also turned to strategies around management to optimise patient outcomes. Considering the need to re-review patients who progress despite therapy, and the impact of an alternative diagnosis, an MDT approach is here and available to help the clinician assess whether disease progression has been evidenced and warrants management decisions, or to help discuss challenging treatment decisions.

Further studies of MDT approaches will be needed to address how they can be successfully utilised in clinical practice.
